# Supplementary material for: Pyruvate Dehydrogenase Kinase 1 inhibition mediated oxidative phosphorylation enhancement in cartilage promotes osteoarthritis progression
Source: BMC Musculoskelet Disord. 2023 Jul 20;24:597. doi: 10.1186/s12891-023-06585-6 (PMC10357736; doi:10.1186/s12891-023-06585-6)
Supplement: Supplementary file 1 — Additional file 1. [file 12891_2023_6585_MOESM1_ESM.zip › BP.Gsea.1653623667859/GOBP_MRNA_TRANSPORT.html]

Details for gene set GOBP\_MRNA\_TRANSPORT[GSEA]

|  || Dataset | 55457\_collapsed\_to\_symbols.cls.cls #OA\_versus\_normal.cls.cls #OA\_versus\_normal\_repos |
| Phenotype | cls.cls#OA\_versus\_normal\_repos |
| Upregulated in class | normal |
| GeneSet | GOBP\_MRNA\_TRANSPORT |
| Enrichment Score (ES) | -0.4485343 |
| Normalized Enrichment Score (NES) | -1.9744383 |
| Nominal p-value | 0.0 |
| FDR q-value | 0.0384315 |
| FWER p-Value | 0.378 |
Table: GSEA Results Summary

  

Fig 1: Enrichment plot: GOBP\_MRNA\_TRANSPORT      
 Profile of the Running ES Score & Positions of GeneSet Members on the Rank Ordered List

  

| SYMBOL | TITLE | RANK IN GENE LIST | RANK METRIC SCORE | RUNNING ES | CORE ENRICHMENT || 1 | EIF5A2 | eukaryotic translation initiation factor 5A2 [Source:HGNC Symbol;Acc:HGNC:3301] | 79 | 0.944 | 0.0182 | No |
| 2 | CETN2 | centrin 2 [Source:HGNC Symbol;Acc:HGNC:1867] | 130 | 0.875 | 0.0368 | No |
| 3 | HHEX | hematopoietically expressed homeobox [Source:HGNC Symbol;Acc:HGNC:4901] | 270 | 0.774 | 0.0459 | No |
| 4 | SEC13 | "SEC13 homolog, nuclear pore and COPII coat complex component [Source:HGNC Symbol;Acc:HGNC:10697]" | 460 | 0.677 | 0.0486 | No |
| 5 | NUP37 | nucleoporin 37 [Source:HGNC Symbol;Acc:HGNC:29929] | 1056 | 0.506 | 0.0151 | No |
| 6 | PCID2 | PCI domain containing 2 [Source:HGNC Symbol;Acc:HGNC:25653] | 1376 | 0.447 | 0.0017 | No |
| 7 | NUP133 | nucleoporin 133 [Source:HGNC Symbol;Acc:HGNC:18016] | 1951 | 0.367 | -0.0337 | No |
| 8 | CETN3 | centrin 3 [Source:HGNC Symbol;Acc:HGNC:1868] | 2114 | 0.348 | -0.0374 | No |
| 9 | THOC7 | THO complex 7 [Source:HGNC Symbol;Acc:HGNC:29874] | 2170 | 0.341 | -0.0328 | No |
| 10 | XPO1 | exportin 1 [Source:HGNC Symbol;Acc:HGNC:12825] | 2477 | 0.305 | -0.0489 | No |
| 11 | IGF2BP3 | insulin like growth factor 2 mRNA binding protein 3 [Source:HGNC Symbol;Acc:HGNC:28868] | 2770 | 0.273 | -0.0647 | No |
| 12 | POLDIP3 | DNA polymerase delta interacting protein 3 [Source:HGNC Symbol;Acc:HGNC:23782] | 2869 | 0.264 | -0.0655 | No |
| 13 | SENP2 | SUMO specific peptidase 2 [Source:HGNC Symbol;Acc:HGNC:23116] | 3040 | 0.246 | -0.0725 | No |
| 14 | PEG10 | paternally expressed 10 [Source:HGNC Symbol;Acc:HGNC:14005] | 3118 | 0.238 | -0.0724 | No |
| 15 | NUP93 | nucleoporin 93 [Source:HGNC Symbol;Acc:HGNC:28958] | 3194 | 0.231 | -0.0723 | No |
| 16 | MVP | major vault protein [Source:HGNC Symbol;Acc:HGNC:7531] | 3511 | 0.201 | -0.0918 | No |
| 17 | ENY2 | ENY2 transcription and export complex 2 subunit [Source:HGNC Symbol;Acc:HGNC:24449] | 3561 | 0.197 | -0.0905 | No |
| 18 | TNKS | tankyrase [Source:HGNC Symbol;Acc:HGNC:11941] | 3622 | 0.192 | -0.0903 | No |
| 19 | SLBP | stem-loop binding protein [Source:HGNC Symbol;Acc:HGNC:10904] | 3703 | 0.185 | -0.0917 | No |
| 20 | MYO1C | myosin IC [Source:HGNC Symbol;Acc:HGNC:7597] | 3730 | 0.183 | -0.0890 | No |
| 21 | NUP155 | nucleoporin 155 [Source:HGNC Symbol;Acc:HGNC:8063] | 3824 | 0.174 | -0.0918 | No |
| 22 | NUP43 | nucleoporin 43 [Source:HGNC Symbol;Acc:HGNC:21182] | 4408 | 0.120 | -0.1343 | No |
| 23 | UPF1 | UPF1 RNA helicase and ATPase [Source:HGNC Symbol;Acc:HGNC:9962] | 4434 | 0.118 | -0.1332 | No |
| 24 | NUP42 | nucleoporin 42 [Source:HGNC Symbol;Acc:HGNC:17010] | 4479 | 0.115 | -0.1337 | No |
| 25 | DDX19B | DEAD-box helicase 19B [Source:HGNC Symbol;Acc:HGNC:2742] | 4500 | 0.113 | -0.1323 | No |
| 26 | NUP54 | nucleoporin 54 [Source:HGNC Symbol;Acc:HGNC:17359] | 4582 | 0.106 | -0.1359 | No |
| 27 | NUP85 | nucleoporin 85 [Source:HGNC Symbol;Acc:HGNC:8734] | 4630 | 0.101 | -0.1370 | No |
| 28 | RAE1 | ribonucleic acid export 1 [Source:HGNC Symbol;Acc:HGNC:9828] | 4697 | 0.096 | -0.1397 | No |
| 29 | MX2 | MX dynamin like GTPase 2 [Source:HGNC Symbol;Acc:HGNC:7533] | 4743 | 0.092 | -0.1408 | No |
| 30 | G3BP2 | G3BP stress granule assembly factor 2 [Source:HGNC Symbol;Acc:HGNC:30291] | 5001 | 0.073 | -0.1590 | No |
| 31 | LUZP4 | leucine zipper protein 4 [Source:HGNC Symbol;Acc:HGNC:24971] | 5009 | 0.072 | -0.1577 | No |
| 32 | KIF5C | kinesin family member 5C [Source:HGNC Symbol;Acc:HGNC:6325] | 5639 | 0.021 | -0.2063 | No |
| 33 | NXF3 | nuclear RNA export factor 3 [Source:HGNC Symbol;Acc:HGNC:8073] | 5648 | 0.020 | -0.2065 | No |
| 34 | TPR | "translocated promoter region, nuclear basket protein [Source:HGNC Symbol;Acc:HGNC:12017]" | 5919 | 0.001 | -0.2275 | No |
| 35 | NUP88 | nucleoporin 88 [Source:HGNC Symbol;Acc:HGNC:8067] | 5976 | -0.003 | -0.2318 | No |
| 36 | GLE1 | GLE1 RNA export mediator [Source:HGNC Symbol;Acc:HGNC:4315] | 6058 | -0.009 | -0.2379 | No |
| 37 | SMG6 | SMG6 nonsense mediated mRNA decay factor [Source:HGNC Symbol;Acc:HGNC:17809] | 6175 | -0.019 | -0.2465 | No |
| 38 | NUP210 | nucleoporin 210 [Source:HGNC Symbol;Acc:HGNC:30052] | 6437 | -0.038 | -0.2659 | No |
| 39 | NUTF2 | nuclear transport factor 2 [Source:HGNC Symbol;Acc:HGNC:13722] | 6536 | -0.045 | -0.2724 | No |
| 40 | NUP62 | nucleoporin 62 [Source:HGNC Symbol;Acc:HGNC:8066] | 6800 | -0.063 | -0.2914 | No |
| 41 | RBM15B | RNA binding motif protein 15B [Source:HGNC Symbol;Acc:HGNC:24303] | 6867 | -0.069 | -0.2948 | No |
| 42 | SETD2 | "SET domain containing 2, histone lysine methyltransferase [Source:HGNC Symbol;Acc:HGNC:18420]" | 6926 | -0.074 | -0.2974 | No |
| 43 | NDC1 | NDC1 transmembrane nucleoporin [Source:HGNC Symbol;Acc:HGNC:25525] | 6943 | -0.075 | -0.2967 | No |
| 44 | AAAS | aladin WD repeat nucleoporin [Source:HGNC Symbol;Acc:HGNC:13666] | 7058 | -0.084 | -0.3034 | No |
| 45 | NUP205 | nucleoporin 205 [Source:HGNC Symbol;Acc:HGNC:18658] | 7080 | -0.086 | -0.3029 | No |
| 46 | CHTOP | chromatin target of PRMT1 [Source:HGNC Symbol;Acc:HGNC:24511] | 7184 | -0.095 | -0.3085 | No |
| 47 | RANBP17 | RAN binding protein 17 [Source:HGNC Symbol;Acc:HGNC:14428] | 7194 | -0.096 | -0.3067 | No |
| 48 | POLR2D | RNA polymerase II subunit D [Source:HGNC Symbol;Acc:HGNC:9191] | 7726 | -0.136 | -0.3447 | No |
| 49 | FMR1 | FMRP translational regulator 1 [Source:HGNC Symbol;Acc:HGNC:3775] | 7944 | -0.151 | -0.3578 | No |
| 50 | MCM3AP | minichromosome maintenance complex component 3 associated protein [Source:HGNC Symbol;Acc:HGNC:6946] | 8077 | -0.161 | -0.3639 | No |
| 51 | LRPPRC | leucine rich pentatricopeptide repeat containing [Source:HGNC Symbol;Acc:HGNC:15714] | 8249 | -0.175 | -0.3728 | No |
| 52 | BICD2 | BICD cargo adaptor 2 [Source:HGNC Symbol;Acc:HGNC:17208] | 8253 | -0.175 | -0.3685 | No |
| 53 | NUP214 | nucleoporin 214 [Source:HGNC Symbol;Acc:HGNC:8064] | 8301 | -0.178 | -0.3676 | No |
| 54 | NCBP2 | nuclear cap binding protein subunit 2 [Source:HGNC Symbol;Acc:HGNC:7659] | 8478 | -0.192 | -0.3764 | No |
| 55 | THOC5 | THO complex 5 [Source:HGNC Symbol;Acc:HGNC:19074] | 8549 | -0.198 | -0.3768 | No |
| 56 | HNRNPA2B1 | heterogeneous nuclear ribonucleoprotein A2/B1 [Source:HGNC Symbol;Acc:HGNC:5033] | 8576 | -0.200 | -0.3736 | No |
| 57 | HNRNPA3 | heterogeneous nuclear ribonucleoprotein A3 [Source:HGNC Symbol;Acc:HGNC:24941] | 8603 | -0.201 | -0.3705 | No |
| 58 | SUPT6H | "SPT6 homolog, histone chaperone and transcription elongation factor [Source:HGNC Symbol;Acc:HGNC:11470]" | 8752 | -0.213 | -0.3766 | No |
| 59 | DDX25 | DEAD-box helicase 25 [Source:HGNC Symbol;Acc:HGNC:18698] | 8815 | -0.218 | -0.3758 | No |
| 60 | UPF3B | UPF3B regulator of nonsense mediated mRNA decay [Source:HGNC Symbol;Acc:HGNC:20439] | 8986 | -0.230 | -0.3831 | No |
| 61 | NCBP1 | nuclear cap binding protein subunit 1 [Source:HGNC Symbol;Acc:HGNC:7658] | 9149 | -0.244 | -0.3895 | No |
| 62 | NUP107 | nucleoporin 107 [Source:HGNC Symbol;Acc:HGNC:29914] | 9274 | -0.253 | -0.3926 | No |
| 63 | NXF2 | nuclear RNA export factor 2 [Source:HGNC Symbol;Acc:HGNC:8072] | 9577 | -0.281 | -0.4090 | No |
| 64 | NXT1 | nuclear transport factor 2 like export factor 1 [Source:HGNC Symbol;Acc:HGNC:15913] | 9983 | -0.319 | -0.4324 | No |
| 65 | SMG5 | SMG5 nonsense mediated mRNA decay factor [Source:HGNC Symbol;Acc:HGNC:24644] | 10190 | -0.340 | -0.4398 | Yes |
| 66 | SRSF1 | serine and arginine rich splicing factor 1 [Source:HGNC Symbol;Acc:HGNC:10780] | 10272 | -0.348 | -0.4371 | Yes |
| 67 | SRSF3 | serine and arginine rich splicing factor 3 [Source:HGNC Symbol;Acc:HGNC:10785] | 10280 | -0.349 | -0.4287 | Yes |
| 68 | ARC | activity regulated cytoskeleton associated protein [Source:HGNC Symbol;Acc:HGNC:648] | 10364 | -0.357 | -0.4259 | Yes |
| 69 | UPF3A | UPF3A regulator of nonsense mediated mRNA decay [Source:HGNC Symbol;Acc:HGNC:20332] | 10455 | -0.368 | -0.4235 | Yes |
| 70 | DHX9 | DExH-box helicase 9 [Source:HGNC Symbol;Acc:HGNC:2750] | 10627 | -0.386 | -0.4269 | Yes |
| 71 | SEH1L | SEH1 like nucleoporin [Source:HGNC Symbol;Acc:HGNC:30379] | 10651 | -0.388 | -0.4187 | Yes |
| 72 | YTHDC1 | YTH domain containing 1 [Source:HGNC Symbol;Acc:HGNC:30626] | 10687 | -0.391 | -0.4113 | Yes |
| 73 | PARP11 | poly(ADP-ribose) polymerase family member 11 [Source:HGNC Symbol;Acc:HGNC:1186] | 10766 | -0.401 | -0.4071 | Yes |
| 74 | NUP98 | nucleoporin 98 and 96 precursor [Source:HGNC Symbol;Acc:HGNC:8068] | 10875 | -0.413 | -0.4049 | Yes |
| 75 | SMG7 | SMG7 nonsense mediated mRNA decay factor [Source:HGNC Symbol;Acc:HGNC:16792] | 10877 | -0.414 | -0.3943 | Yes |
| 76 | SRSF7 | serine and arginine rich splicing factor 7 [Source:HGNC Symbol;Acc:HGNC:10789] | 10930 | -0.422 | -0.3875 | Yes |
| 77 | NUP160 | nucleoporin 160 [Source:HGNC Symbol;Acc:HGNC:18017] | 11100 | -0.444 | -0.3892 | Yes |
| 78 | EIF5A | eukaryotic translation initiation factor 5A [Source:HGNC Symbol;Acc:HGNC:3300] | 11127 | -0.446 | -0.3798 | Yes |
| 79 | NUP58 | nucleoporin 58 [Source:HGNC Symbol;Acc:HGNC:20261] | 11132 | -0.447 | -0.3685 | Yes |
| 80 | HSF1 | heat shock transcription factor 1 [Source:HGNC Symbol;Acc:HGNC:5224] | 11189 | -0.456 | -0.3612 | Yes |
| 81 | KHSRP | KH-type splicing regulatory protein [Source:HGNC Symbol;Acc:HGNC:6316] | 11285 | -0.469 | -0.3565 | Yes |
| 82 | NUP153 | nucleoporin 153 [Source:HGNC Symbol;Acc:HGNC:8062] | 11307 | -0.472 | -0.3459 | Yes |
| 83 | DDX39A | DExD-box helicase 39A [Source:HGNC Symbol;Acc:HGNC:17821] | 11359 | -0.480 | -0.3375 | Yes |
| 84 | NCBP3 | nuclear cap binding subunit 3 [Source:HGNC Symbol;Acc:HGNC:24612] | 11502 | -0.501 | -0.3357 | Yes |
| 85 | ZC3H3 | zinc finger CCCH-type containing 3 [Source:HGNC Symbol;Acc:HGNC:28972] | 11571 | -0.511 | -0.3278 | Yes |
| 86 | ZC3H11A | zinc finger CCCH-type containing 11A [Source:HGNC Symbol;Acc:HGNC:29093] | 11780 | -0.546 | -0.3300 | Yes |
| 87 | DDX19A | DEAD-box helicase 19A [Source:HGNC Symbol;Acc:HGNC:25628] | 11821 | -0.553 | -0.3189 | Yes |
| 88 | NUP188 | nucleoporin 188 [Source:HGNC Symbol;Acc:HGNC:17859] | 11877 | -0.567 | -0.3086 | Yes |
| 89 | THOC6 | THO complex 6 [Source:HGNC Symbol;Acc:HGNC:28369] | 11918 | -0.576 | -0.2968 | Yes |
| 90 | AGFG1 | ArfGAP with FG repeats 1 [Source:HGNC Symbol;Acc:HGNC:5175] | 12035 | -0.602 | -0.2904 | Yes |
| 91 | NUP50 | nucleoporin 50 [Source:HGNC Symbol;Acc:HGNC:8065] | 12127 | -0.626 | -0.2813 | Yes |
| 92 | NPIPA1 | nuclear pore complex interacting protein family member A1 [Source:HGNC Symbol;Acc:HGNC:7909] | 12133 | -0.627 | -0.2655 | Yes |
| 93 | POM121 | POM121 transmembrane nucleoporin [Source:HGNC Symbol;Acc:HGNC:19702] | 12146 | -0.631 | -0.2502 | Yes |
| 94 | UPF2 | UPF2 regulator of nonsense mediated mRNA decay [Source:HGNC Symbol;Acc:HGNC:17854] | 12150 | -0.631 | -0.2342 | Yes |
| 95 | IGF2BP2 | insulin like growth factor 2 mRNA binding protein 2 [Source:HGNC Symbol;Acc:HGNC:28867] | 12244 | -0.657 | -0.2245 | Yes |
| 96 | SMG1 | SMG1 nonsense mediated mRNA decay associated PI3K related kinase [Source:HGNC Symbol;Acc:HGNC:30045] | 12268 | -0.668 | -0.2091 | Yes |
| 97 | AHCTF1 | AT-hook containing transcription factor 1 [Source:HGNC Symbol;Acc:HGNC:24618] | 12312 | -0.681 | -0.1949 | Yes |
| 98 | QKI | "QKI, KH domain containing RNA binding [Source:HGNC Symbol;Acc:HGNC:21100]" | 12340 | -0.688 | -0.1792 | Yes |
| 99 | HNRNPA1 | heterogeneous nuclear ribonucleoprotein A1 [Source:HGNC Symbol;Acc:HGNC:5031] | 12356 | -0.693 | -0.1625 | Yes |
| 100 | NXF1 | nuclear RNA export factor 1 [Source:HGNC Symbol;Acc:HGNC:8071] | 12410 | -0.710 | -0.1483 | Yes |
| 101 | PABPN1 | poly(A) binding protein nuclear 1 [Source:HGNC Symbol;Acc:HGNC:8565] | 12461 | -0.730 | -0.1334 | Yes |
| 102 | THOC1 | THO complex 1 [Source:HGNC Symbol;Acc:HGNC:19070] | 12503 | -0.749 | -0.1173 | Yes |
| 103 | RANBP2 | RAN binding protein 2 [Source:HGNC Symbol;Acc:HGNC:9848] | 12609 | -0.804 | -0.1048 | Yes |
| 104 | AKAP8L | A-kinase anchoring protein 8 like [Source:HGNC Symbol;Acc:HGNC:29857] | 12665 | -0.836 | -0.0875 | Yes |
| 105 | THOC2 | THO complex 2 [Source:HGNC Symbol;Acc:HGNC:19073] | 12691 | -0.855 | -0.0674 | Yes |
| 106 | DDX39B | DExD-box helicase 39B [Source:HGNC Symbol;Acc:HGNC:13917] | 12783 | -0.941 | -0.0502 | Yes |
| 107 | ZFP36 | ZFP36 ring finger protein [Source:HGNC Symbol;Acc:HGNC:12862] | 12846 | -1.052 | -0.0279 | Yes |
| 108 | ZFP36L1 | ZFP36 ring finger protein like 1 [Source:HGNC Symbol;Acc:HGNC:1107] | 12888 | -1.244 | 0.0009 | Yes |
Table: GSEA details [plain text format]

  

Fig 2: GOBP\_MRNA\_TRANSPORT      
 Blue-Pink O' Gram in the Space of the Analyzed GeneSet

  

Fig 3: GOBP\_MRNA\_TRANSPORT: Random ES distribution      
 Gene set null distribution of ES for **GOBP\_MRNA\_TRANSPORT**
